# Relationship between Dispositional Mindfulness, Psychological Health, and Diet Quality among Healthy Midlife Adults

**DOI:** 10.3390/nu12113414

**Published:** 2020-11-06

**Authors:** Shannon D. Donofry, Kirk I. Erickson, Michele D. Levine, Peter J. Gianaros, Matthew F. Muldoon, Stephen B. Manuck

**Affiliations:** 1 Department of Psychology, University of Pittsburgh, Pittsburgh, PA 15260, USA; kiericks@pitt.edu (K.I.E.); levinem@upmc.edu (M.D.L.); gianaros@pitt.edu (P.J.G.); manuck@pitt.edu (S.B.M.); 2 The Center for the Neural Basis of Cognition, Pittsburgh, PA 15260, USA; 3 Department of Psychiatry, University of Pittsburgh School of Medicine, Pittsburgh, PA 15260, USA; 4 Department of Obstetrics, Gynecology, and Reproductive Sciences, University of Pittsburgh, Pittsburgh, PA 15260, USA; 5 Heart and Vascular Institute, School of Medicine, University of Pittsburgh, Pittsburgh, PA 15260, USA; mfm10@pitt.edu

**Keywords:** dispositional mindfulness, depressive symptoms, diet quality, healthy eating index

## Abstract

Mindfulness, a practice of non-judgmental awareness of present experience, has been associated with reduced eating psychopathology and emotion-driven eating. However, it remains unclear whether mindfulness relates to diet quality. Thus, the purpose of this study was to examine whether dispositional mindfulness is associated with diet quality and to explore psychological factors relating dispositional mindfulness to diet quality. Community-dwelling adults (*N* = 406; *M_age_* = 43.19, *SD* = 7.26; *M_body mass index [BMI]_* = 27.08, *SD* = 5.28; 52% female) completed ratings of dispositional mindfulness, depressive symptoms, perceived stress, positive affect (PA), and negative affect (NA). Dietary intake was assessed using the Block Food Frequency Questionnaire, from which the 2015 Healthy Eating Index was derived. Analyses were conducted using the “lavaan” package in R with bias-corrected bootstrapped confidence intervals (BootCI). Age, sex, race, education, and BMI were entered as covariates in all models. Higher dispositional mindfulness was associated with higher diet quality (*β* = 0.11, *p* = 0.03), and this effect was mediated through lower depressive symptoms (indirect effect *β* = 0.06, *p* = 0.02, BootCI = 0.104–1.42, *p* = 0.03). Dispositional mindfulness was negatively correlated with perceived stress (*β* = −0.31, *p* < 0.01) and NA (*β* = −0.43, *p* < 0.01), as well as positively correlated with PA (*β* = −0.26, *p* < 0.01). However, these factors were unrelated to diet quality. These cross-sectional data provide initial evidence that dispositional mindfulness relates to diet quality among midlife adults, an effect that may be explained in part by less depressive symptomatology. Given that lifestyle behaviors in midlife are leading determinants of risk for cardiovascular disease and neurocognitive impairment in late life, interventions to enhance mindfulness in midlife may mitigate disease risk. Additional research assessing the impact of mindfulness interventions on diet quality are warranted.

## 1. Introduction

Poor diet quality is one of the leading modifiable risk factors for mortality worldwide, increasing susceptibility for numerous adverse physical and psychological health outcomes, including cardiovascular disease [1], diabetes [2], dementia [3,4], some forms of cancer [5,6], and major depression [7]. These negative health effects translate into significant social and economic burden. It is estimated that unhealthy dietary patterns contribute to 18.2% of all costs associated with treatment of cardiometabolic disease in the U.S., which translates to $50.4 billion annually [8]. Yet despite the clear benefits of adopting a healthy diet, most Americans do not consume a diet that meets federal recommendations [9]. Further, existing approaches to improving dietary adherence have primarily been developed for use in clinical or at-risk populations [10,11,12]. These interventions are typically delivered via time- and resource intensive means (e.g., individual, clinic-based intervention) and thus require significant individual commitment to change [13]. To improve the health and well-being of the general population and to mitigate risk for chronic disease, there is a pressing need to identify barriers to healthy eating and to develop novel strategies to enhance adherence to dietary guidelines.

Among the complex and interacting factors that have been shown to influence dietary intake patterns [14], the capacity to engage self-regulation skills in response to cognitive, social and emotional triggers for eating has been identified as one of the most important predictors of eating and weight management outcomes [15]. Psychological factors, such as perceived stress, depressed mood, and negative affect, increase vulnerability for lapses in self-regulation of eating. For instance, depressive symptoms have been associated with reduced likelihood of maintaining weight loss [16], and eating as a means of regulating mood is predictive of weight regain following a weight loss attempt [17]. Experimental induction of negative affect or stress has been associated with altered attentional processing of palatable food cues [18,19,20,21,22], and affect-driven shifts in attention predict subsequent eating behavior [18,23,24,25,26,27] and weight gain [28]. Daily life stress is also related to higher calorie intake from foods high in sugar and fat [29,30]. Together, these data suggest that mood and stress may impair control of eating by disrupting self-regulation. Interventions that enhance self-regulation may therefore improve eating behaviors and compliance with dietary recommendations.

Mindfulness-based practices can enhance several aspects of self-regulation relevant to the control of eating behavior [31] and therefore hold promise as a strategy for improving eating patterns and dietary quality. Mindfulness involves maintaining moment to moment awareness of and openness to current experiences, including cognitions, emotions, and physical sensations, and resistance of any immediate urges to react to those experiences [32]. Cultivation of mindfulness skills is thought to disrupt engagement in automatic or habitual responses to one’s experiences, allowing for more deliberative, goal-concordant decision making. Evidence from both observational and intervention research suggests that mindfulness is associated with improved psychological and physical health outcomes [7,33,34,35]. For instance, higher dispositional mindfulness, a trait-like tendency to be aware of present moment experiences in daily life, is related to lower rumination, depressive symptoms, and anxiety [36]. Mindfulness may also lead to reduced cardiovascular disease risk [34]. A recent meta-analysis of mindfulness-based interventions documented a beneficial effect of mindfulness practice on body mass index [35], and observational studies have shown that dispositional mindfulness is related to other indicators of cardiovascular health, such as glucose regulation [37] and blood pressure [38]. Individuals high in dispositional mindfulness also report engaging in less emotional and external eating behaviors [39,40], suggesting that mindfulness may reduce affect- and cue-driven eating. Moreover, mindfulness-based interventions have been shown to reduce weight-related eating psychopathology, such as binge eating [35,41], as well as to reduce high calorie food intake following exposure to appetitive cues [42]. Indeed, there are now several mindfulness-based interventions for the treatment of disordered eating, including Acceptance and Commitment Therapy for Binge Eating Disorder and obesity [43,44,45], and Mindfulness-Based Eating Awareness Training (MB-EAT) [46,47,48]. Given the benefits of mindfulness practices for eating psychopathology, it is possible that these practices may be effective for improving other aspects of eating behavior, including dietary quality.

Despite these findings, the majority of research examining the relationship between mindfulness and eating behavior has focused on eating psychopathology, rather than overall energy intake or dietary quality. Several prior cross-sectional studies observed a positive relationship between dispositional mindfulness and healthy dietary habits [49,50,51], and participation in an 8-week Mindfulness-Based Stress Reduction (MBSR) intervention was associated with reduced intake of fast food and desserts [52]. However, all of these studies used abbreviated questionnaires to obtain information about dietary intake, the majority of which were not designed to assess dietary intake but rather to assess health behaviors more broadly. Further, dietary intake and dietary quality are only modestly correlated (*r* = 0.23 [53]) and thus represent distinct aspects of overall food consumption. Therefore, although these findings suggest that mindfulness may relate to dietary intake, additional research using high-quality assessments of dietary intake and dietary quality are needed to fully characterize the relationship between mindfulness and food intake patterns among healthy adults. Further, while it is clear that mindfulness improves psychological health and that psychological health impacts eating behavior and weight, it is unclear as to whether psychological factors, such as depressed mood or perceived stress, explain associations between mindfulness and eating outcomes. Accordingly, the purpose of the present study was to evaluate the relationship between dispositional mindfulness, psychological health, and dietary quality in a community sample of healthy midlife adults free of medical or psychological conditions. It was hypothesized that individuals reporting higher dispositional mindfulness would also report consuming a higher quality diet and that this relationship would be mediated by psychological factors, such as depressive symptomatology, perceived stress, and affect.

## 2. Materials and Methods

### 2.1. Participants

Participants included 490 community dwelling adults aged 30–54 from the second wave of the Adult Health and Behavior Project (AHAB-II), a registry of behavioral and biological correlates of chronic disease risk. Recruitment occurred through mass mailings of letters to individuals randomly selected from voter registration and other public domain lists in Western Pennsylvania between February 2008 and October 2011. To be eligible, participants had to be in generally good health and working at least 25 h per week outside of the home (due to a sub-study investigating the relationship between occupational stress and cardiovascular outcomes). Exclusion criteria included a clinical history of neurologic illness, cardiovascular disease, cancer treatment within the previous year, chronic hepatitis, renal failure, any neurological or cerebrovascular disorder, lung disease requiring drug treatment, stage 2 hypertension (systolic/diastolic blood pressure ≥ 160/100 mmHg), alcohol consumption ≥5 portions 3–4 times per week, schizophrenia, or other psychoses, or shift work. Volunteers were also excluded if they reported current use of insulin, glucocorticoid, lipid-lowering, antiarrhythmic, psychotropic, or prescription weight-loss medications or taking fish oil supplements. Women were excluded if pregnant or lactating. Data collection occurred over the course of several laboratory visits, and informed consent was obtained in accordance with the guidelines of the University of Pittsburgh Institutional Review Board.

### 2.2. Assessments

#### 2.2.1. Dispositional Mindfulness

Participants completed the Mindful Attention Awareness Scale (MAAS) [54], a 15-item questionnaire that assesses core features of mindfulness, including openness to and awareness of present moment experiences. For each item, participants were asked to rate the frequency at which they were distracted, unaware, or on “automatic pilot” during daily tasks or experiences (e.g., “I could be experiencing some emotion and not be conscious of it until some time later”). Items were rated on a 0 (“Almost Always”) to 6 (“Almost Never”) point Likert scale and responses were averaged to form a mean total score ranging from 0–6. Higher scores are indicative of a greater tendency to be mindful in daily life. The MAAS has been validated in a number of populations, including university undergraduate students [55,56,57], healthy community adults [54], and in clinical populations [58,59]. Estimates of scale reliability have been satisfactory (Cronbach’s α = 0.82) [54], including in the present sample (Cronbach’s α = 0.89). Mindfulness-based interventions have been shown to improve MAAS scores [60], suggesting that the scale possesses construct validity. Further, higher MAAS scores have been associated with higher self-regulation [61], as well as lower self-reported stress and depressive symptomatology [58,61] and impulsivity [62].

#### 2.2.2. Depressive Symptoms

Depressive symptoms were assessed using the Center for Epidemiologic Studies-Depression Scale (CES-D) [63], a self-report measure of the frequency of 20 common depressive symptoms rated along a 0 (“Rarely or none of the time”) to 3 (“Most or all of the time”) on the Likert scale. Responses are summed to yield a total symptom score (maximum possible score of 60) with higher scores reflecting more severe depressive symptoms. The CES-D has demonstrated adequate reliability and validity in a number of populations [64].

#### 2.2.3. Perceived Stress

The 10-item version of the Perceived Stress Scale (PSS) [65] was administered to assess experiences of daily life stress. The PSS is an instrument on which respondents use a 0 (“Never”) to 4 (“Very Often”)-point Likert scale to rate the degree to which daily life events are perceived to be uncontrollable, unpredictable, or unmanageable. Responses are summed to form a total score (maximum possible score of 40). This scale has been shown to exhibit satisfactory reliability (Cronbach’s α = 0.85) and validity [66].

#### 2.2.4. Positive and Negative Affect

To evaluate individual differences in trait positive affect (PA) and negative affect (NA), participants were administered the Positive Affect Negative Affect Schedule-Expanded Form (PANAS-X; see Reference [67]. The PANAS-X features 60 adjectives that describe distinct emotional states corresponding to several affective domains, including PA (e.g., “inspired”) and NA (e.g., “irritable”). Respondents are asked to rate the extent to which they tend to experience each emotional state using a 1 (“very slightly or not at all”) to 5 (“extremely”) point Likert scale. Sub-domain scores are obtained by summing responses to the subset of items belonging to a given domain. Both PA and NA domains are comprised of 10 items each, with scores ranging from 10–50 for each domain. Higher scores represent higher levels of PA and NA. The PANAS-X PA and NA scales have been shown to exhibit acceptable reliability (PA Cronbach’s α = 0.89; NA Cronbach’s α = 0.85 [68]; and validity [67,68,69].

#### 2.2.5. Dietary Intake and Quality

Dietary intake was evaluated using the 2005 version of the Block Food Frequency Questionnaire (FFQ), a 110-item inventory that estimates usual and customary intake of a number of nutrients and food items [70]. The list of food items was developed based on dietary intake data obtained from the 1998–2002 National Health and Nutrition Examination Survey (NHANES), with the selected food items contributing to over 90% of the calories and 17 macro- and micronutrients reported by NHANES respondents. AHAB-II participants were asked to recall frequency of intake of each food item using a 1 (“never”) to 9 (“every day”) point Likert scale, and to estimate usual portion size consumed for intake occurring within the prior four months. To improve accuracy of portion size recall, participants viewed photographs of reference food items of various portion sizes displayed on a plate. A series of “adjustment” questions about how foods were prepared were included to more accurately assess fat intake (e.g., type and fat content of ground meat consumed). Responses were then used to calculate estimates of nutrient and food group intake according to the U.S. Department of AgricultureFood and Nutrient Database for Dietary Studies (FNDDS), version 1. The Block FFQ has moderate reliability and validity [71,72].

Nutrient and food intake data obtained from the FFQ were then used to calculate the 2015 version of the healthy eating index (HEI), a measure of dietary quality developed to quantify the degree to which an individual’s dietary intake patterns conform to the recommendations put forth in the 2015–2020 Dietary Guidelines for Americans [73]. The HEI is comprised of 13 subcomponents, nine of which are categorized as adequacy subcomponents and 4 of which are categorized as moderation components. Adequacy subcomponents capture intake of food groups and nutrients for which higher intake is desirable, such as whole fruits and total vegetables, while moderation subcomponents reflect intake of food groups or nutrients for which it is recommended that intake be limited (e.g., added sugars). For each subcomponent, higher scores reflect a pattern of healthier intake for a given subcomponent. Thus, higher scores on adequacy subcomponents reflect higher intakes while higher scores on moderation subcomponents reflect lower intakes. Scores on each subcomponent are summed to form a total HEI score ranging from 0–100, with higher scores indicating dietary intake more closely aligned with the Dietary Guidelines for Americans. Estimates obtained from several large nationally representative surveys suggest that the average HEI-2015 score for Americans is 56.6 with a range of 32.6 to 81.2 [53]. Correlations between HEI scores and total caloric intake were observed to be low (*r* < 0.25), suggesting that these two aspects of dietary intake provide unique information about consumption patterns [53] and may independently relate to individual differences in health outcomes linked with diet. Higher HEI-2015 scores have been associated with lower all-cause mortality and reduced risk of mortality, specifically from cardiovascular disease, Type II Diabetes, and cancer [53,74,75].

### 2.3. Statistical Approach

Prior to hypothesis testing, all data were examined to determine missingness, identify extreme values, and confirm that the data structure met analytic assumptions. To examine total, direct, and indirect effects of MAAS scores on HEI-2015 scores, a path model was tested specifying a direct pathway with MAAS scores predicting HEI-2015 scores, four indirect pathways operating through each hypothesized mediator (CES-D, PSS, NA, and PA scores), and a total effect pathway modeling the combined effect of both direct and indirect paths. Each indirect pathway was modeled as the product of the regression of MAAS scores on a given mediation variable and the regression of the mediation variable on the HEI-2015 scores. Residual covariances among the four mediating variables were included in the model. Non-parametric bootstrapping with 5000 simulations was performed for estimates of direct, indirect, and total effects. Because the chi-square test of model fit is highly sensitive to minor sources of misfit between estimated models and observed data, model fit was evaluated using multiple alternative fit statistics (Brown, 2006), including comparative fit index (CFI; 0.95 or above indicative of good fit), root mean square error of approximation (RMSEA; 0.05 or below indicative of good fit), and standardized root mean square residual (SRMR; 0.08 or below indicative of good fit). Age, sex, race (white vs. non-white), education, and body mass index (BMI) were included as covariates. Path modeling was performed in R version 4.0.2 using the ‘lavaan’ package [76].

#### Exploratory Analyses

To further examine the relationship between mindfulness and dietary intake patterns, an exploratory path model was constructed to assess the direct effect of MAAS scores on each HEI subcomponent, as well as the indirect effect of MAAS scores on each subcomponent, operating through the proposed psychological mediators. In addition, alternative mediation models were tested to determine whether the theoretical models adopted in the primary analyses provided a superior statistical fit to the data. Please see supplementary material for details of exploratory analyses with HEI-2015 subcomponent scores and alternative mediation models.

## 3. Results

### 3.1. Sample Characteristics

AHAB-II participants who did not complete the MAAS were excluded from all analyses (*n =* 84), yielding a final sample of 406 individuals. Individuals with missing MAAS data were not significantly different from those retained for analyses in terms of race, CES-D scores, PSS scores, PA, NA, BMI, caloric intake, or HEI scores. However, compared to individuals who did not complete the MAAS, those with available MAAS data were older (*OR* = 1.059, *p* < 0.01), reported having fewer years of education (*OR* = 0.664, *p* < 0.01), and were less likely to be female (*OR* = 0.543, *p* = 0.03).

**Figure 1:**
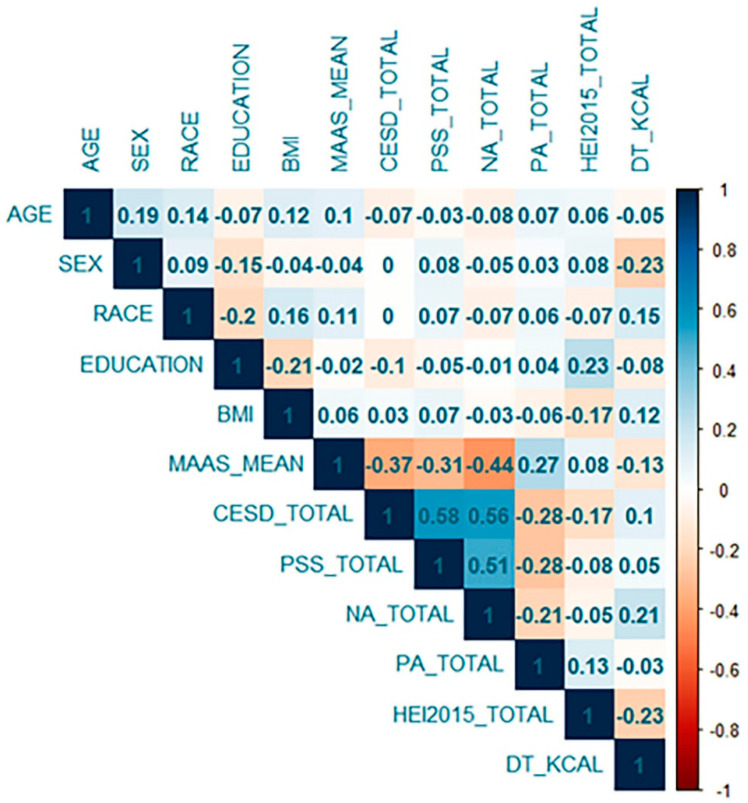
Correlation matrix among all demographic and clinical characteristics. Note: BMI = body mass index; MAAS = Mindful Awareness and Attention Scale; PSS = Perceived Stress Scale; CES-D = Center for Epidemiologic Studies Depression Scale; PANAS = Positive Affect Negative Affect Schedule; NA = negative affect; PA = positive affect; HEI = Healthy Eating Index; DT_KCAL = Daily total intake in kilocalories. Matrix created in R using the ‘corrplot’ package.

Mean MAAS scores were 4.28 (*SD* = 0.74), comparable to scores obtained in prior investigations among healthy adults [54,55]. Participants reported depressive symptoms in the mild range (*M* = 8.93, *SD* = 8.25, range = 0–45), with 72 individuals (17.7%) scoring above the clinical cutoff of 16. Ratings of perceived stress were in the moderate range of severity (*M* = 15.89, *SD* = 3.96, range = 6–29). Higher age (*β* = 0.10, *p* = 0.04) and minority race (*β* = 0.13, *p* = 0.01) were associated with higher MAAS scores, while sex, education, and BMI were not significantly related to MAAS scores. HEI-2015 scores were higher among women (*β* = 0.11, *p* = 0.01) and those with higher education (*β* = 0.21, *p* < 0.01), as well as were negatively associated with BMI (*β* = −0.12, *p* = 0.01). Higher education was related to lower CES-D scores (*β* = −0.10, *p* = 0.03). Age was negatively correlated with negative affect scores (*β* = −0.09, *p* = 0.04), while BMI was negatively correlated with positive affect scores (*β* = −0.09, *p* = 0.04). Demographic characteristics and BMI were not significantly associated with PSS scores. Table 1 contains the demographic and clinical characteristics of the sample. Correlations among all variables of interest are depicted in Figure 1.

### 3.2. Total and Mediating Effects of Dispositional Mindfulness on Dietary Quality

**Figure 2:**
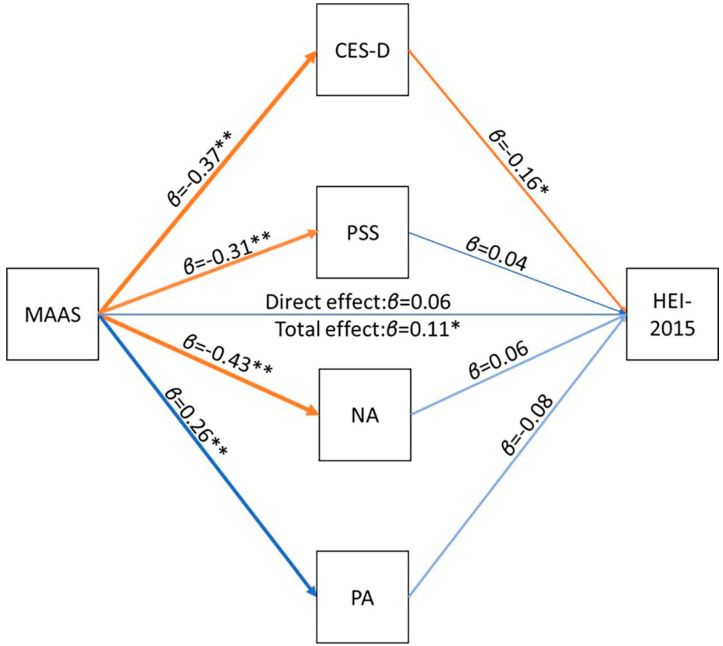
Diagram of path model of the effect of MAAS scores on HEI-2015 total scores. Note: MAAS = Mindful Awareness and Attention Scale; PSS = Perceived Stress Scale; CES-D = Center for Epidemiologic Studies Depression Scale; PANAS = Positive Affect Negative Affect Schedule; NA = negative affect; PA = positive affect; HEI = Healthy Eating Index. * *p* < 0.05 ** *p* < 0.01.

Overall fit of the path model was satisfactory (χ² (20) = 29.717 *p =* 0.07; RMSEA = 0.035, 90% confidence interval =0.000–0.059; CFI = 0.982; SRMR = 0.030). Results of the model are depicted in Figure 2. As predicted, higher dispositional mindfulness scores were associated with lower CES-D (*β* = −0.37, *p* < 0.01), PSS (*β* = −0.31, *p* < 0.01), and NA scores (*β* = 0.43, *p* < 0.01), and higher PA scores (*β* = 0.26, *p* < 0.01). Higher MAAS scores were also significantly associated with higher dietary quality (*β* = 0.11, *p* = 0.03), but this effect was not significant when adjusting for scores on the psychological measures (*β* = 0.06, *p* = 0.24). CES-D scores significantly mediated the relationship between MAAS scores and HEI-2015 total scores (effect of CES-D scores on HEI-2015 scores: *β* = −0.16, *p* = 0.02; magnitude of indirect effect: *β* = 0.06, *p* = 0.02), with higher dispositional mindfulness being related to higher diet quality through lower depressive symptoms. In contrast, none of the other psychological factors were related to HEI-2015 total scores and, therefore, did not act as statistical mediators of the relationship between dispositional mindfulness and dietary quality (*p* > 0.15). CES-D scores also significantly mediated the effect of MAAS scores on total fruit (*β* = 0.06, *p* < 0.01), whole fruit (*β* = 0.06, *p* < 0.01), and whole grain (*β* = 0.05, *p* = 0.01) subcomponent scores, and marginally mediated the effect of MAAS scores on added sugar intake (*β* = 0.04, *p* = 0.05; see Supplementary materials, including Table S1 for additional information). Finally, there were no significant mediation effects observed in the alternative mediation models tested, suggesting that the theoretical model adopted in the primary analyses was statistically superior (see Table S2 for detailed results from these models).

## 4. Discussion

The purpose of the present study was to examine whether dispositional mindfulness was related to dietary quality among healthy midlife adults, and to explore potential psychological factors that might explain this relationship. Consistent with our hypotheses, higher dispositional mindfulness was related to consuming a higher quality diet, a relationship which persisted after adjusting for the potentially confounding effects of demographic characteristics and BMI. In particular, higher dispositional mindfulness was associated with higher reported intakes of vegetables, fruit, and whole grains, and lower intakes of added sugars. Two recent meta-analyses have documented a beneficial effect of mindfulness practices on problem eating behaviors, such as emotional eating, dietary restriction, and binge eating [35,41]. The present findings extend these results to include potential benefits to dietary intake and quality among generally healthy community adults. This suggests that mindfulness-based interventions may help to promote health behavior change in the general population, potentially in conjunction with other primary prevention strategies already in place. It will be important to further examine the impact of mindfulness on dietary intake and quality using well-designed randomized controlled trials of mindfulness-based interventions, such as MBSR and MB-EAT.

Importantly, we also found that the relationship between dispositional mindfulness and dietary quality was statistically mediated by lower depressive symptoms, suggesting that depressed mood may be a modifiable pathway by which mindfulness is related to dietary intake. Symptoms of depression, such as anhedonia, lethargy, and indecisiveness, may make it difficult to put forth the effort and planning necessary to obtain and prepare healthy foods. Depression has also been associated with reduced interoceptive accuracy, or the ability to detect changes in internal bodily sensations [77,78]. Changes in bodily sensations in response to external events are the peripheral component of emotional reactivity, and the degree to which an individual recognizes when these changes are occurring relates to the intensity of emotional experience, as well the ability to regulate emotional responses [79]. Lowered interoceptive accuracy in depression may therefore suggest that individuals who are experiencing depressive symptoms may not be as perceptive of visceral sensations relevant to mood (e.g., heartbeat) or eating (e.g., satiety signals, such as stomach distension), which may impede their ability to detect when an internal state has changed and to adapt behavior accordingly. In addition, preliminary evidence suggests that mindfulness interventions may improve interoceptive accuracy and self-awareness [80,81,82,83] and that improvements in interoceptive accuracy may facilitate improvements in self-regulation of emotion and behavior (e.g., Reference [46]. Mindfulness may also reduce emotional reactivity to distressing or uncomfortable situations [84,85], which may reduce urges to eat in response to negative emotions or increase the ability to regulate these urges when they arise. Further research is necessary to explore these and other potential mechanisms linking mindfulness to dietary intake and quality.

Finally, individuals reporting higher dispositional mindfulness also reported less perceived stress and negative affect, and higher positive affect, a finding that converges with prior research [36], as well as with evidence from clinical trials of mindfulness-based interventions [86,87]. Interestingly, these psychological factors did not predict dietary quality. This is in contrast to some prior research, which found evidence to suggest that dietary quality is inversely associated with factors, such as stress and negative affect [88,89]. Differences in the way in which depressive symptoms and negative affect are conceptualized may explain why depressive symptom severity was the only psychological factor to statistically mediate the relationship between dispositional mindfulness and dietary quality. Indeed, NA and PA as measured by the PANAS are broad constructs that include a wide range of emotional experiences, while the CES-D evaluates various symptoms associated with one type of negative emotional experience, depression. It is possible that some negative emotions have more of an impact on dietary intake than others, and the summation across multiple negative emotions in the NA scale may obscure this.

### Limitations and Future Directions for Research

To our knowledge, the present study is one of the first and the largest to examine whether dispositional mindfulness is related to dietary quality among healthy community adults and to explore possible psychological factors by which mindfulness may relate to diet. Further, our analyses accounted for several factors known to influence psychological health, dietary intake, and accuracy of dietary reporting (age, sex, education, and BMI; Reference [90,91,92,93]). Nevertheless, there are a number of important limitations that should be taken into account when interpreting the present findings. First, these data are cross-sectional and therefore cannot resolve whether there is a causal relationship between mindfulness and dietary quality. It will be imperative to explore the effectiveness of mindfulness-based interventions for improving dietary intake and quality using a clinical trial design. Second, participants in the AHAB-II sample may not be fully representative of the general population. Participants on average had more years of education than is typical in the general population, and HEI scores were about 10 points higher than the reported average for Americans (58.7; Reference [9]). Relatedly, because the sample was predominantly white, it was not possible to examine racial and ethnic differences in psychological health and diet quality. Given the alarming racial health disparities in the United States, this is a critical area for future research. Third, dietary intake is notoriously difficult to assess, and food recall instruments, such as the FFQ, used in the present study are subject to a number of biases that negatively impact accuracy of recall [94]. Additional research using other dietary assessment methods, such as a 24-h recall interview format, is needed to more firmly establish the relationship between mindfulness and dietary quality. A fourth limitation is that we only explored whether psychological factors, such as mood and stress, mediated the relationship between dispositional mindfulness and dietary quality. Although this is an important contribution to the literature, there are other potentially independent pathways through which mindfulness might relate to dietary intake and quality that were not measured in the present study. For instance, mindfulness has been related to performance on tests of higher-order executive functions relevant to self-regulation, eating behavior, and obesity, including attentional control [95] and response inhibition [96]. Further research is necessary to explore other potential mechanistic pathways through which mindfulness may influence dietary intake and quality. Finally, there is debate about the utility of self-report assessments of mindfulness and to what extent these measures capture the construct of mindfulness [97]. However, evidence suggests that scores on the MAAS improve with mindfulness training [60], providing some confidence that this measure possesses adequate construct validity.

## 5. Conclusions

The present findings contribute to the growing literature demonstrating the benefits of mindfulness to health and suggest a potential psychological construct by which mindfulness may relate to health behaviors, such as dietary intake. We demonstrated a relationship between mindfulness, depressive symptoms, and dietary quality among healthy midlife adults free of medical or psychological conditions, providing initial evidence that mindfulness practices are not only beneficial for individuals with a diagnosed illness but may also be an effective approach to disease prevention.

## Figures and Tables

**Table 1:** Demographic and clinical characteristics of the sample (*N* = 406).

	Mean *(SD)*
Age (years)	43.19 (7.26)
BMI (kg/m^2^)	27.08 (5.79)
MAAS average score	4.28 (0.74)
PSS score	15.89 (3.96)
CES-D score	8.93 (8.25)
PANAS NA score	15.57 (5.17)
PANAS PA score	34.1 (6.69)
Total caloric intake (kilocalories)	1901 (894.5)
HEI 2015 Total Score	67.58 (10.59)
	*N* (%)
Sex (F)	211 (51.97)
Education Level	
No High School diploma	1 (0.20)
GED	4 (0.98)
High School diploma	23 (5.66)
Technical training	19 (4.68)
Some college	42 (10.34)
Associates degree	39 (9.61)
Bachelor’s degree	148 (36.45)
Master’s degree	90 (22.17)
Doctoral degree	40 (9.85)
Race	
White	328 (80.79)
Black or African American	72 (17.73)
Asian	2 (0.49)
Multi-racial	2 (0.49)
Other	2 (0.49)

Note: Race was coded as white (0) vs. non-white (1) for all analyses. BMI = body mass index; MAAS = Mindful Awareness and Attention Scale; PSS = Perceived Stress Scale; CES-D = Center for Epidemiologic Studies Depression Scale; PANAS = Positive Affect Negative Affect Schedule; NA = negative affect; PA = positive affect; HEI = Healthy Eating Index.

## Supplementary Materials

The following are available online at https://www.mdpi.com/2072-6643/12/11/3414/s1, Table S1. HEI-2015 subcomponent scores and scoring standards. Table S2. Outcomes from alternative mediation models.
