# Supplementary material for: Relationship between Dispositional Mindfulness, Psychological Health, and Diet Quality among Healthy Midlife Adults
Source: Nutrients. 2020 Nov 6;12(11):3414. doi: 10.3390/nu12113414 (PMC7695007; doi:10.3390/nu12113414)
Supplement: Supplementary file 1 [file nutrients-12-03414-s001.pdf]

**Table S1.** HEI-2015 subcomponent scores and scoring standards.

| Subcomponent | *M* (*SD*) | Maximum Score | Criteria for Maximum |
|---|---|---|---|
| Adequacy subcomponents | | | |
| Total fruits | 3.61 (1.50) | 5 | ≥0.8 cup equivalent per 1000 kilocalories |
| Whole fruits | 3.95 (1.42) | 5 | ≥0.4 cup equivalent per 1000 kilocalories |
| Total vegetables | 4.23 (1.02) | 5 | ≥1.1 cup equivalent per 1000 kilocalories |
| Greens and beans | 4.31 (1.21) | 5 | ≥0.2 cup equivalent per 1000 kilocalories |
| Whole grains | 4.55 (2.74) | 10 | ≥1.5-ounce equivalent per 1000 kilocalories |
| Dairy | 5.63 (2.49) | 10 | ≥1.3 cup equivalent per 1000 kilocalories |
| Total protein | 4.56 (0.73) | 5 | ≥2.5-ounce equivalent per 1000 kilocalories |
| Seafood and plant proteins | 4.42 (1.00) | 5 | ≥0.8-ounce equivalent per 1000 kilocalories |
| [1] Fatty acids | 6.38 (2.71) | 10 | (PUFAs + MUFAs)/SFAs ≥ 2.5 |
| Moderation subcomponents | | | |
| Refined grains | 8.10 (2.12) | 10 | ≤1.8-ounce equivalent per 1000 kilocalories |
| Sodium | 3.99 (2.49) | 10 | ≤1.1 g per 1000 kilocalories |
| Added sugars | 7.86 (2.32) | 10 | ≤6.5% of energy |
| Saturated fats | 5.99 (2.84) | 10 | ≤8% of energy |

[1] Ratio of poly- and mono-unsaturated fatty acids (PUFAs and MUFAs) to saturated fatty acids (SFAs).

Note: Scoring standards are from the USDA Center for Nutrition Policy and Promotion (https://www.fns.usda.gov/how-hei-scored).

Exploratory analysis of HEI-2015 subcomponent scores. To further examine the relationship between mindfulness and dietary intake patterns, an exploratory path model was tested with the 13 HEI subcomponent scores as outcome variables. Because depressive symptoms were the only psychological factor to mediate the relationship between MAAS scores and total HEI-2015 scores, the exploratory path model examining the direct and mediating effect of MAAS scores on HEI subcomponent scores only included CES-D scores as a mediator. Specifically, a path model was constructed to assess the direct effect of MAAS scores on each HEI subcomponent, as well as the indirect effect of MAAS scores, on each subcomponent operating through CES-D scores. Age, sex, race, education, and BMI were included as covariates in the model. Path modeling was performed in R using the 'lavaan' package [76]. Table S1 includes HEI subcomponent scores and scoring standards.

Alternative mediation models. To determine the robustness of the target mediation model (MAAS → CES-D → HEI-2015), from which we observed a significant mediating effect of CES-D scores on the relationship between MAAS scores and HEI-2015 total scores, three alternative variants of the target mediation model were tested. In the first variant, CES-D scores were modeled as the X variable, MAAS scores as the M variable, and HEI-2015 scores as the Y variable. The second model variant was a reverse mediation test of the target model, and included MAAS scores as the X variable, HEI-2015 scores as the M variable, and CES-D scores as the Y variable. In the third model variant, CES-D scores were included as the X variable, HEI-2015 scores were included as the M variable, and MAAS scores were included as the Y variable. For each mediating variable, the fitted mediator model (e.g., CESD scores predicting MAAS scores) and total effects model (e.g., effect of CESD scores on HEI scores controlling for MAAS scores) were entered as inputs for causal mediation analysis using the 'mediation' package for R [98]. This produces an estimate of the average causal mediation effect (ACME) of a given independent variable operating through a given mediator. Non-parametric bootstrapping with 5000 simulations was performed to generate bias-corrected and accelerated confidence intervals (BootCI) for estimates of indirect, total, and direct effects.

**Supplementary Results:** HEI-2015 subcomponent scores. Results from the path model indicated that there was a significant direct effect of MAAS scores on total vegetable subcomponent scores ($\beta = 0.12$, $p = 0.02$), with higher MAAS scores being associated with greater reported intake of foods included in this subcomponent. CES-D scores significantly mediated the effect of MAAS scores on total fruit ($\beta$=0.06, $p$<0.01), whole fruit ($\beta = 0.06$, $p < 0.01$), and whole grain ($\beta = 0.05$, $p = 0.01$) subcomponent scores, and marginally mediated the effect of MAAS scores on added sugar intake ($\beta = 0.04$, $p = 0.05$). There were no other direct or mediating effects of MAAS scores on HEI subcomponent scores (*p*s > 0.06).

**Table S2.** Outcomes from alternative mediation models.

| | **Effect** | ***p*-Value** |
|---|---|---|
| Model 1: CES-D Scores → MAAS Scores → HEI-2015 Scores | | |
| ACME | −0.264 | 0.26 |
| ADE | −1.174 | 0.05 |
| Total effect | −1.438 | **0.01** |
| Model 2: MAAS Scores → HEI-2015 Scores → CES-D Scores | | |
| ACME | −0.014 | 0.08 |
| ADE | −0.050 | **<0.01** |
| Total effect | −0.518 | **<0.01** |
| Model 3: CES-D Scores → MAAS Scores → HEI-2015 Scores | | |
| ACME | −0.006 | 0.27 |
| ADE | −0.289 | **<0.01** |
| Total effect | −0.295 | **<0.01** |

Note: Bolded values indicate significant effects. CES-D = Center for Epidemiologic Studies Depression Scale; MAAS = Mindful Attention Awareness Scale; HEI = Healthy Eating Index; ACME = Average Causal Mediation Effect; ADE = Average Direct Effect.

Alternative mediation models. Table S2 provides detailed results from each of the alternative mediation models tested. There were no significant mediation effects detected for any of the alternative models. Significant direct and total effects of MAAS scores on CES-D scores (alternative model two) and of CES-D scores on HEI-2015 total scores (alternative model three) were observed. Although the cross-sectional nature of these data preclude strong statements about which model is most plausible, these results provide statistical support for the theoretical model adopted and tested in our primary analyses.
